# Beyond the Surface: Tracing the Evolution of Inflammatory Mechanism in Depression through Bibliometric Analysis

**DOI:** 10.2174/0118715303325541241024060229

**Published:** 2025-01-08

**Authors:** Zhang-Yang Xu, Ting Zhang, Hong Gong, Hong Zheng, Mei-Shan Liu, Bing-Hang Guo, Yun-Xia Wang, Wen-Jun Su

**Affiliations:** 1 Department of Stress Medicine, Faculty of Psychology, Second Military Medical University, Shanghai 200433, China;; 2 Department of Nautical Psychology, Faculty of Psychology, Second Military Medical University, Shanghai 200433, China;; 3 Department of Developmental Neuropsychology, School of Medical Psychology, Army Medical University Third Military Medical University, Chongqing 400038, China;; 4 The Battalion 2 of Cadet Brigade, School of Basic Medicine, Second Military Medical University, Shanghai 200433, China;; 5 The Battalion 3 of Cadet Brigade, School of Basic Medicine, Second Military Medical University, Shanghai 200433, China

**Keywords:** Depression, bibliometric, inflammation, citeSpace, neuroimmunology, WoSCC

## Abstract

**Background:**

Depression is a common mental illness that has become a major economic burden worldwide. Recently, increasing evidence has highlighted the inflammatory mechanism of depression. In order to understand the research status of this field, this study used the bibliometric analysis method to overview the research content and progress, as well as analyze the development trend and limitations.

**Methods:**

In this study, articles and reviews were included in the specific search strategy. The matched papers were exported from the Web of Science database. CiteSpace 6.3 R1 and Bibliometrix (R package) were utilized to generate bibliometric and knowledge maps.

**Results:**

A total of 25,063 articles were included in this study. The number of publications in this field has gradually increased, especially in recent years. These papers come from 156 countries, led by the United States and China mainland. The leading research institution is the University of Toronto (Canada). Brain Behavior and Immunity is the journal with the most publications and the most frequently co-cited journals. Among 91,100 authors, Maes M has the most publications and co-citations. According to the keywords burst and co-cited reference analysis, the hotspots in the field in recent years include kynurenine, c-reactive protein, neuroinflammation, and gut microbiota.

**Conclusion:**

Although abundant academic achievements have been made on the inflammatory mechanism of depression, there is still a long way to go before these research results can be applied to clinical practice. Strengthening international academic exchanges and cooperation may promote breakthroughs in this field.

## 1 INTRODUCTION

Depression (major depressive disorder, MDD) is a mood disorder characterized by depressed mood, loss of interest, feelings of worthlessness or guilt, and other symptoms grouped into emotional, neurovegetative, and neurocognitive domains [1-3]. It may cause cognitive dysfunction in patients and even lead to suicide in severe cases. According to estimates, about 280 million people worldwide are suffering from depressive disorders [4]. Given its high occurrence and severe impact, it has been regarded as one of the most prevalent and debilitating diseases in the world. Moreover, due to medical isolation, loss of loved ones, uncertainty, and many other problems during the COVID-19 pandemic, the incidence of depression increased by 27.6% worldwide in 2020 [5, 6]. Collectively, depression brings a heavy burden to the healthcare and economic system and is thereby listed as one of the leading causes of the global disease burden [7-10].

At present, research on the pathogenesis of depression is mainly related to monoamine transmitter deficiency, hypothalamic-pituitary-adrenal (HPA) axis dysfunction, neural plasticity abnormality, inflammatory imbalance, gut microbiota dysbiosis, *etc* [2, 11-15]. Specifically, inflammation has recently been suggested to play an important role in psychiatric disorders, including mood disorders, such as depression [16-18]. The cytokine hypothesis of depression was first proposed by Smith [19] in 1991 in the form of the “macrophage theory of depression”, and further demonstrated and elucidated by Maes *et al.* [20] in the early 1990s. During the past three decades, emerging studies have shown that long-term exposure to increasing low-grade inflammation leads to changes in neurotransmitters and neural circuits associated with depressive symptoms, which may also affect the efficacy and efficiency of antidepressant treatment [21-25]. Therefore, the relationship between depression and inflammation has become one of the current research hotspots in the pathophysiology of depression. Considering that numerous works of literature have reported the relationship in this field, it seems difficult and inefficient to obtain information about researchers, institutions, journals, topics, and keywords by summarizing and refining the overall research content only through a literature review. Therefore, a more convenient and intuitive way to access this information is needed.

Bibliometrics is a discipline that uses mathematical and statistical methods to quantitatively analyze previously published articles, books, and other communication media [26]. It can visually describe the knowledge resources possessed by people in a certain period of time and the relevant characteristics of their carriers, and it can draw, analyze, and demonstrate scientific and technical knowledge as well as their internal connections [27, 28]. In general, the statistical scope of bibliometrics mainly includes the contents most directly related to the literature, such as authors, keywords, countries, institutions, *etc*. Besides, through word frequency analysis, citation analysis, and co-citation analysis, it can reveal the research status of a certain field within a certain period, which is very helpful to analyze and comprehend the development trend and direction of specific research fields.

In line with this, this study aimed to classify and summarize the works of literature published from 1990 to 2023 and to overview the inflammatory mechanism of depression from a qualitative and quantitative perspective by combining the literature with visual analysis software. The research contents mainly include:

1. Analysis of the number and growth trends of publications in the field of the relationship between depression and inflammation.
2. Investigation of countries/regions, institutional, collaboration network of countries/regions, and institution.
3. Investigation of authors, journal publications, and citations.
4. Exploration of keywords, research hotspots and trends.
5. Analysis of the co-cited reference clustering, burst value, and structural variation.

Briefly, the purpose of this research was to explore the progress path of the inflammatory mechanism of depression and point out the research hotspots, development trends, existing insufficiencies, and future direction in this field. To the best of our knowledge, the use of bibliometric methods to systematically analyze and summarize the inflammatory mechanism of depression has not been observed. Thus, the results will contribute to the study of the relationship between depression and inflammation and provide a reliable basis for selecting journals, collaborators, and frontiers for future research, thereby facilitating research on the pathogenesis, diagnosis, prevention, and treatment of depression.

## 2 METHODS

### 2.1 Data Collection

In this paper, works of literature were collected from the Science Citation Index Expanded and Social Science Citation Index of Web of Science Core Collection (WOSCC), which include the following database: Science Citation Index Expanded (SCI-EXPANDED); Social Sciences Citation Index (SSCI); Arts & Humanities Citation Index (A&HCI); Conference Proceedings Citation Index- Science (CPCI-S); Conference Proceedings Citation Index- Social Science & Humanities (CPCI-SSH); Book Citation Index– Science (BKCI-S); Book Citation Index– Social Sciences & Humanities (BKCI-SSH); Emerging Sources Citation Index (ESCI); Current Chemical Reactions (CCR-EXPANDED); Index Chemicus (IC). The data were retrieved from 1990 to 2023(12-30). The search terms were as follows: “(TS= (“inflammation” OR “cytokines” OR “neuroinflammation” OR “inflammatory” OR “pro-inflammatory” OR “anti-inflammatory”)) AND TS= (“depression” OR “depressive”)”. Only the documents of article or review type were included, while other document types, such as meeting abstracts, letters, book reviews, and corrections, were excluded. Finally, a total of 25,063 papers were included in this study. The literature searching and identifying flowchart is shown in Fig. (**1**). Then, the relevant literature retrieved by this method was exported in “plain text” file format and sorted in a “.txt” format. The exported records included “full records and references cited”.

### 2.2 Data Analysis

All valid papers retrieved from the WOSCC database were converted to Microsoft Excel, Bibliometrix (R package), and CiteSpace software to perform visual analysis. CiteSpace, a Java application widely used for bibliometric analysis, was created by Professor Chen Chaomei (College of Computing and Informatics, Drexel University, Philadelphia, PA, USA) [29]. In this study, CiteSpace 6.3 R1 knowledge graph visualization software was used for data analysis. Based on the co-citation analysis theory and pathfinding network algorithm, an econometric analysis of literature in a certain field was performed, and a series of visual knowledge maps were drawn by analyzing data information to explore knowledge inflection points and evolution routes in a certain field. On this basis, the research direction and development trend were pointed out to provide research frontier direction for researchers. Microsoft Excel 2016 was used to sort out the exported data and draw tables. According to previously published methods [30], the time slicing segment was set to 1990.01 to 2023.12, and the year per slice was set to 1 year, while g-index was set to k= 20. Node type was an institution, author, keyword, and country. Besides, the construction mode was set as a pathfinder and pruning sliced networks. According to the abovementioned settings, visual analysis was conducted to generate knowledge maps.

## 3 RESULTS

### 3.1 Annual Publication

A total of 25,063 records were included; the number of publications by year is presented in Fig. (**2**). From 1990 to 2006, the number of publications rose from 9 references to 293 references. From 2007 to 2011, the annual growth rate of the number of published papers increased steadily. From 2012 to 2022, the number of published scientific papers on the relationship between depression and inflammation expanded, and the total number of publications in 2023 reached 2,536.

### 3.2 Distribution of Countries/Regions

A total of 25,063 papers were published by 156 countries/regions, accounting for 66.95% of 233 countries/regions in the world, which indicates that this topic is gaining extensive attention worldwide. Fig. (**3**) presents the geographical distribution of these 156 countries/regions. As shown in this map, the top 10 countries with the most publications are United States of America (USA, publication: 7775), People's Republic of China (Peoples’ R China/China mainland, publication: 4525), United Kingdom (UK, publication: 1889), Canada (publication: 1635), Germany (publication: 1628), Italy (publication: 1438), Australia (publication: 1284), Brazil (publication: 1169), Netherlands (publication: 1021), and France (publication: 791). In an overall view, these papers are mainly distributed in the USA, the People's Republic of China, and some European countries/regions, such as the UK, Germany, Netherlands, Italy, and France. Developed countries/regions and large developing countries/regions are the main forces in the research field of the inflammatory mechanism of depression, occupying the primary position.

### 3.3 Collaboration Network of Countries/Regions and Institutions

The scientific collaboration network of countries/regions and institutions in Fig. (**4**) clearly shows the leading countries/regions and research institutions in this field and their collaborations.

Table **1** (left panel) lists the top 10 countries/regions ranked by collaboration frequency. In terms of collaboration frequency, the top 10 countries/regions are the USA, People's R China, the UK, Canada, Germany, Italy, Australia, Brazil, Netherlands, and France.

Since 1990 to 2023, a total of 29,223 institutions have published papers in the field. Table **1** (right panel) lists the top 10 institutions based on publication. The top 10 institutions with the most international cooperation are University of Toronto (Canada), King’s College London (UK), University of California, Los Angeles (USA), Deakin University (Australia), University of Melbourne (Australia), Emory University (USA), Harvard Medical School (USA), University of Pittsburgh (USA), University College London (UK) and Ohio State University (USA). Fig. (**5**) shows 884 nodes and 2545 connections with a network density of 0.0065. Each node represents one institution, and the lines represent the link between nodes. Combined with publication and centrality in the cooperation network, the University of Toronto (publication: 445, centrality: 0.04) and Kings College London (publication: 431, centrality: 0.04) seem to be the major institutions. Besides, the University of California is showing strong momentum within this field, with its burst index of 27.34.

### 3.4 Authors and Co-Cited Authors

A total of 91,100 authors have published papers on the topic of the inflammatory mechanism of depression. As shown in Table **2**, the top 10 authors who published the most on this topic are Maes M (publication: 608), Berk M (publication: 350), Zhang Y (publication: 307), Pariante CM (publication:271), Miller AH (publication: 251), Mcintyre RS (publication: 214), Carvalho AF (publication: 192), Dantzer R (publication: 157), Irwin MR (publication: 155) and Galecki P (publication: 149). In terms of the frequency of co-citation, the top 10 authors are Maes M (frequency: 4026; centrality: 0.16), Dantzer R (frequency: 3521; centrality: 0.13), Miller AH (frequency: 2989; centrality: 0.02), Raison CL (frequency: 2696; centrality: 0.01), Dowlati Y (frequency: 2081; centrality: 0.01), Capuron L (frequency: 1558; centrality: 0.01), Howren MB (frequency: 1249; centrality: 0.00), Kessler RC (frequency: 1216; centrality: 0.00), Felger JC (frequency: 981; centrality: 0.00) and Beck AT (frequency: 953; centrality: 0.10). Collectively, it seems that the most influential authors in the field are Maes M from Deakin University, Dantzer R from University of Illinois, Miller AH from Emory University School of Medicine and Raison CL from University of Wisconsin-Madison (previous in Emory University School of Medicine). They publish not only in large quantities but also in high quality.

### 3.5 Journals And Co-Cited Journals

Papers related to the inflammatory mechanisms of depression were published in 3,106 journals. Table **3** lists the top 10 journals and co-cited journals on the association between depression and inflammation. The Journal of Brain Behavior and Immunity (publication: 974, 3.43%) has the largest number of publications, followed by the Journal of Affective Disorders (500, 1.76%) and Biological Psychiatry (498, 1.75%), each with more than 400 publications. In terms of the influence of these journals, as evaluated by the Impact Factor (IF), all the top 10 journals have an IF higher than 3.0. Among them, Brain Behavior and Immunity (IF=15.1) ranks first, followed by the Journal of Neuroinflammation with an IF of 6.6. Specifically, the Journal of Brain Behavior and Immunity, founded in 1987, is the official journal of the Psychoneuroimmunology Research Society (PNIRS). According to its aims and scope, the innovative journal is interested in basic, experimental, and clinical studies dealing with behavioral, neural, endocrine, and immune system interactions in humans and animals. Similarly, the Journal of Neuroinflammation is a fully open-access journal focusing on interactions of the immune system (especially the innate immune system) with the nervous system.

Co-citation analysis is designed to assess the relationship between articles. To some extent, the frequency of co-citations of a journal reflects its influence and importance in the specific research field. Among the 2096 co-cited journals, 214 journals were cited over 1,000 times. As is shown in Table **3** (right panel), Brain Behavior and Immunity (10757) is the most frequently co-cited journal, followed by Biological Psychiatry (10334) and Plos One (9615). Among the top 10 co-cited journals, the Lancet has the highest IF (168.9), followed by Nature (IF=64.8) and Brain Behavior and Immunity (IF=15.1). Furthermore, according to the Journal Citation Indicator (JCI) rank list in Journal Citation Reports (JCR) 2023, nine out of the ten top co-cited journals are distributed in the Q1 quartile, except for Plos One.

The dual-map overlay of journals indicates the research foundation and application status in the field of inflammatory mechanisms of depression. The field of “application” refers to the main application field of current research on the inflammatory mechanism of depression, while “research basis” means the corresponding research that provides theories, methods, technologies, and other supporting fields. Regarding the citation relationship, the former refers to all citing papers, while the latter refers to all cited papers. Fig. (**6**) shows the distribution in the field of “inflammatory mechanism of depression”, with the left and right sides representing the application and research basis of the field, respectively. The extended ellipse with journal clustering as the core represents the number of literature in this cluster. Further, the longer horizontal axis represents more authors in the cluster, and the longer vertical axis indicates more publications. Specifically, the largest visible oval is “Molecular, Biology, Genetics”, which suggests that the journals publishing relevant papers are primarily located in these categories. Also, there are six main citation paths in Fig. (**6**), including 3 orange paths and 3 green paths. The green paths indicate that studies published in categories of Molecular/Biology/Genetics, Health/Nursing/Medicine journals, and Psychology/Education/Social are cited for journals from Medicine/Medical/Clinical categories. Meanwhile, the orange paths demonstrate that studies published in journals of the Molecular/Biology/Immunology category mostly cite papers from Molecular/Biology/Genetics, Health/Nursing/Medicine, and Psychology/Education/Social fields.

### 3.6 Keyword Burst Value Analysis

The keyword burst value analysis identifies the hot keywords that attract the most attention of peer researchers within a certain period of time. As is depicted in Fig. (**7**), the top 25 keywords with the strongest burst values are well summarized. Regarding the entire period from 1990 to 2023, “major depression” (strength: 225.27) has the highest burst strength, followed by “tumor necrosis factor” (strength: 178.52), “necrosis factor α” (strength: 104.25), “pituitary adrenal axis ” (strength: 92.77), “myocardial infarction” (strength: 89.45) and “coronary heart disease” (strength: 89.36). More specifically, during the 1990s, this field focuses on “tumor necrosis factor”, “rat”,, “interferon γ”, “septic shock”, “central nervous system”, “factor α”, “corticotropin releasing hormone”, “expression”, “nitric oxide”, “interleukin 6”, “pituitary adrenal axis”, and “cytokine”. In the 21st century, the main keywords changed to “cytokine production”, “interferon α”, “psychological stress”, “myocardial infarction”, “coronary heart disease”, “cardiovascular disease”, “risk factor”, and “coronary artery disease”. It is worth noting that keywords that emerged in recent years, including “mortality” (strength: 39.94), “induced sickness behavior” (strength: 37.4), and “gut microbiota” (strength: 53.11), have maintained a high burst value so far, which may be the hot topics for investigators in recent years.

### 3.7 Co-cited Reference Clustering and Time Evolution Analysis

The co-citation relationship is defined by the simultaneous citation of two papers in the third paper. Literature co-citation analysis is an effective method for detecting the structure and evolutionary path of a specific research field. In order to better reflect the temporal characteristics of clustering effects, this study uses a log-likelihood test algorithm to extract nominal terms from keywords in the cited literature and name clusters. The complete mean silhouette of the clustering report of co-cited references is 0.89, and the overall modularity Q value is 0.75, indicating that the clustering effect is efficient and reliable and that the characteristics and definitions of each subdomain are obvious. As shown in Fig. (**8**), publications on the inflammatory mechanisms of depression are divided into 16 clusters, a vertically descending order showed cluster size, and cluster labels were obtained using the log-likelihood ratio (LLR). The largest cluster is “inflammation” (#0, size: 331), followed by clusters of “cytokines” (#1, size: 322), “neuroinflammation” (#2, size: 321), “c-reactive protein” (#3, size: 314), “bipolar disorder” (#4, size: 278), “gut microbiota” (#5, size: 197).

As shown in Fig. (**9**), the number of documents in each cluster is clearly displayed in the timeline view. The more articles in the cluster, the more important the cluster field becomes. The time span of documents in each category can further suggest their temporal characteristics. In the early stages of this research field, especially before 2000, scholars mainly focused on inflammation (#0), coronary artery disease (#6), haptoglobin (#8), and heart failure (#10). At the beginning of this century, from 2000 to 2013, cytokines (#1) and bipolar disorder (#4) became hot topics in research. Subsequently, from 2013 to 2020, a variety of studies gradually focused on neuroimmunology and inflammatory biomarkers, such as neuroinflammation (#2), c-reactive protein (#3), kynurenine (#7), and dietary inflammatory index (#13). In recent years, the main research directions of academia have turned to inflammatory bowel disease (#9), COVID-19 (#12), and gut microbiota (#5). Combined with the keywords analysis, this evolution not only reflects the researchers' points of interest but also demonstrates the continuous deepening and expansion of the inflammatory mechanism of depression.

### 3.8 Co-cited Reference Burst Value and SVA

The analysis of the burst value of co-cited references shows that publications with a high frequency of citations in a particular period are more interested in research on the topic. The top 25 co-cited references with the strongest burst value on the inflammatory mechanism of depression from 1990 to 2023 are summarized in Fig. (**10**). Unsurprisingly, most of the top co-cited references come from the field of Neuropsychoimmunology. In general, these references began to burst in 2005. Among them, the publication “Dowlati Y, 2010, BIOL PSYCHIAT ” (strength: 260.11) had the highest burst strength, followed by the five references with burst strength above 150, including “Miller AH, 2016, NAT REV IMMUNOL ” (strength: 248.34), “Miller AH, 2009, Biol Psychia ” (strength: 201.49), “Howren MB, 2009, Psychosom Med, ” (strength: 176.99), and “Raison CL, 2006, Trends Immunol ” (strength: 152.89). The average duration of the burst value is about 7-8 years. It is worth noting that there are 4 articles, including “Kohler CA, 2017, Acta Psychiat Scand” (strength: 115.04, 2018-2023), “Malhi GS, 2018, LANCET ” (strength: 105.45, 2021-2023), “Troubat R, 2021, Eur J Neurosc” (strength: 81.96, 2021-2023), “Osimo EF, 2020, Brain Behav Immun,” (strength: 81.56, 2021-2023), that remained influential until 2023.

Structural variation analysis (SVA) was conducted to determine the ability of references to establish extraordinary or unexpected connections between different clusters and to detect potential landmark studies in the field of inflammatory mechanisms of depression. Table **4** lists the top 6 references for SVA, including 3 clinical studies, 2 reviews, and 1 animal study. Interestingly, four of them were published in 2001, while the other two were published in 2008. According to the modularity change rate, the top 6 references are all above 96, which indicates that these papers have played an important role in the knowledge evolution of research in this field. Specifically, in the article published in Psychological Medicine in 2001, Maes *et al.* [31] reported that the reduction in the availability of plasma tryptophan in pregnant women from the end of term to the early puerperium was related to immune activation. In view of the fact that the activation of the inflammatory response system accompanied depression, Maes [32] studied the immunomodulatory effect of antidepressants in the review published in Human Psychopharmacology in 2001. He pointed out that antidepressants have negative immunomodulatory effects, and this effect might be one of the mechanisms of their antidepressant effects. In the clinical trial published in the New England Journal of Medicine in 2001, Musselman *et al.* [33] proposed that paroxetine preconditioning might be an effective strategy to reduce IFN-α-related depression in the treatment of patients with malignant melanoma. In another clinical research published in Cytokine in 2001, Kudoh and collaborators [34] investigated the inflammatory cytokine response of patients with chronic depression during abdominal surgery and found that the response of IL-6 to surgical trauma depended on the clinical state of depression. When the time came to 2008, Godbout *et al.* [35] reported for the first time in animal research that the age-related reactivity of the brain cytokine system could play a pathophysiological role in the increase of the incidence rate of depression in the elderly. This research was published in Neuropsychopharmacology. The sixth article is a review published on CNS Spectrum in 2008. McNally and colleagues [36] discussed the interaction between inflammation and glutamate dysfunction in the pathophysiology of depression. They combined the classic monoaminergic, glutamatergic, and neurotrophic hypotheses and proposed a possible mechanism model of inflammation-induced depression rather than overturning them and seeking a new framework.

## 4 DISCUSSION

To the best of our knowledge, this is the first bibliometric analysis overviewing the research progress of the inflammatory mechanism of depression. Since the proposal of the “macrophage theory of depression”, a total of 29,223 institutions in 156 countries/regions have contributed to this research field and published a total of 25,063 articles during the period 1990-2023. Unsurprisingly, the number of scientific papers on this topic has increased annually. Although these papers are mainly from institutions and researchers in developed countries/regions and large developing countries/regions, the strengthening of global cooperation has promoted the rapid development of this research field. In addition, these studies are usually published in professional journals in the categories of immunology and psychiatry. The analysis based on keywords suggests that the hotspots have shifted from systemic inflammation, especially cytokine synthesis and secretion, to central-peripheral crosstalk, such as neuroinflammation and gut-brain axis, as well as the comorbidity studies. Co-citation and time evolution analysis also demonstrate that kynurenine, c-reactive protein, neuroinflammation, and gut microbiota may be promising research directions in the next few years.

Obviously, the cumulative papers show that an increasing number of studies focus on the underlying inflammatory mechanism of depression. Regarding resource and distribution, research papers in this field mainly come from the USA, mainland China, the UK, Germany, Canada, and some other countries/regions in North America, Europe, and Asia-Pacific areas. Although the prevalence of depression in these countries/regions is not the highest [5, 37], the comprehensive power of these countries is relatively strong, especially the economic, cultural, and scientific background [38], and their attention to mental health issues are important factors affecting the development and trend of depression research. Additionally, close cooperation between institutions and researchers from different countries/regions is a key factor in promoting the development of the inflammatory mechanism of depression. Among them, the United States leads in the number of published papers and the frequency of cooperation. It also has a high centrality in the national/regional cooperation network, which fully demonstrates the important position of the United States in this research field. Developed countries such as the UK, Germany, and Canada also have high publications and high centrality in their cooperation networks. As for research institutions, the University of Toronto (Canada), King's College London (UK), and the University of California, Los Angeles (USA) rank in the top 3 in cooperation frequency.

Meanwhile, Emory University (USA) has a relatively high burst index in addition to its high cooperation frequency and centrality. Furthermore, mainland China is also an important contributor, ranking second in terms of the number of published papers and having the highest burst index. This shows that although mainland China started late in this field, it has developed very rapidly, which is inseparable from the promotion brought by the good scientific research environment and financial support. These results coincided with another study overviewing research on major depression in China [39]. It can be estimated that if this trend continues, China will become a leading country in this research field in the near future. However, it is worth noting that mainland China has a relatively low centrality, which reflects that research cooperation between mainland China and other countries/regions is still limited and has insufficient influence [39]. Therefore, China should seek and strengthen global cooperation and exploration to achieve breakthroughs and further development in this field.

In the analysis of contributing authors and co-cited authors, we identified that Maes M from Deakin University not only published the largest number of papers but also had a high quality of these studies, thereby ranking first in both aspects. Besides, in another analysis of journals and co-cited journals, we find that among 4022 journals that published studies on the relationship between inflammation and depression, the top 10 journals contributed about one-eighth (13.82%, 3465/25,063) of research papers. These studies are usually published in some professional journals in the categories of immunology and psychiatry, such as Brain Behavior and Immunity, Journal of Affective Disorders, and Psychneuroendocrinology. Besides, the most frequently co-cited journals are Biological Psychiatry and Brain Behavior and Immunity.

As for the interrelationship among these studies, they cover a wide range of research fields, such as cellular, molecular, environmental, social, nutritional, clinical, biological, and educational aspects. They are closely related to each other, and there are some regular patterns. Firstly, the inflammatory mechanism of depression has a wide range of applications and rich achievements in providing theoretical, methodological, technical, and other supportive research bases. Secondly, the research on the inflammatory mechanism of depression is of great value in medical/clinical applications. Thirdly, basic medical research usually comes from problems raised in clinical practice and will eventually be applied to solve clinical problems. These patterns indicate that the progress of the inflammatory mechanism of depression is not limited to the field of psychiatry but also shows a trend of diversified interdisciplinary development. As reported in 1987, McDonald *et al.* [40]conducted a clinical trial on the use of interferon-α (IFN-α) in the treatment of hepatitis B. The results showed that participants who received IFN-α had a significant increase in psychiatric symptoms, including depressive-like symptoms, some of which were severe enough to require urgent psychiatric treatment. Since then, emerging studies have begun to explore the potential mechanism. Although not all depressive symptoms are caused by inflammation, the inflammatory process is proven to be implicated in the pathophysiology of depression [41]. Exogenous administration of lipopolysaccharide (LPS), a critical component of bacterial endotoxin, as well as cytokines like Interleukin-1β (IL-1β), have been widely used to establish rodent depression models for research use [42, 43]. Furthermore, cytokine inhibitors like infliximab, a tumor necrosis factor (TNF-α) antagonist, showed an antidepressant effect among depressed who had high levels of inflammation at pretreatment [44]. In general, clinical evidence supporting the close relationship between inflammation and depression mainly includes the following: (1) Aberrant levels of proinflammatory or anti-inflammatory cytokines, particularly the chronic low-grade inflammation associated with depressive symptoms [45], are commonly observed in MDD patients [46, 47]. (2) Inflammation-related diseases, including autoimmune diseases [48-51], coronary artery disease [52] and cancer [53], are highly comorbid with depression [54]. (3) Multiple randomized controlled clinical trials have consistently demonstrated the efficacy of psychotherapy in alleviating both depressive symptoms associated with Inflammation-related diseases and the symptoms of these diseases themselves [55-57]. (4) Experimental endotoxemia induced by LPS injection [58, 59] or administration of exogenous pro-inflammatory cytokines [60] can lead to sickness behavior or mood disturbance in humans. (5) Antidepressants and other effective treatments for depression can reduce the level of inflammatory cytokines in depressive patients to a certain extent [61]. (6) The level of peripheral inflammatory factors is related to the severity and prognosis of depression [62-64]. (7) Anti-inflammatory therapy is effective in improving depressive symptoms, whether used as monotherapy [65, 66] or as add-on therapy [67], so it is proposed to be an effective and promising treatment for depression [68-70] (Table **5**).

In order to explore the research hotspots and trends in this field, this paper also analyzes and summarizes the keywords in different periods. In the early 1990s, the association between clinical depression and immunity attracted the attention of many researchers. It was also reported that the administration of cytokines like IFN-α induced anxious and depressive symptoms like lack of motivation, difficulty concentrating, and fatigue [71-85]. So, the keywords for the early research in this field focused on inflammatory processes and immune components. Afterward, it was found that there was a close relationship between depression and cardiovascular disease. Depression was not only an independent risk factor for cardiovascular disease but also a risk factor for adverse cardiovascular outcomes in patients with cardiovascular diseases [86-90]. Subsequently, it was summarized that cardiovascular disease and depression might share a common pathology, including increased sympathetic nervous system activity, hypothalamic pituitary adrenal axis hyperactivity, and inflammation [51, 91-93]. In recent years, researchers have established animal models and simulated depressive symptoms with depressive-like behaviors [94-100], thus deepening the understanding of the inflammatory mechanism and strengthening preclinical research and clinical application [101-103].

On the one hand, the conservative view that the central nervous system was an immune-privileged organ has been broken, and the role and mechanism of neuroinflammation in depression have attracted much attention from researchers [43, 104]. Visualization and quantification of neuroinflammation in depressive patients by positron emission tomography (PET) is also becoming a popular research direction [7, 105-108]. On the other hand, emerging works of literature have discussed the relationship between depression and gut microbiota from the perspectives of metabolism, nerves, and immunity [15, 109-112]. Similar conclusions can be drawn through co-cited reference clustering and burst value analysis. The COVID-19 pandemic has witnessed a substantial surge in the prevalence of mental health disorders, particularly depression. The pathogenesis of COVID-19-induced depression may involve the initiation of peripheral inflammatory response by the coronavirus, resulting in disruption of the blood-brain barrier and subsequent activation of neuroinflammatory processes. These cascading events ultimately lead to synaptic damage or neuronal loss, thereby manifesting as depressive symptoms or cognitive impairment [113]. Furthermore, COVID-19 survivors exhibit persistent psychopathology and neurocognitive impairment [114, 115]. However, the administration of anti-inflammatory interventions shows promise for effectively alleviating mood disturbances and cognitive deficits among individuals with long-COVID [116, 117].

The main strengths of the present bibliometric research are that it intuitively answers which national/regional institutions and researchers have published the most papers on the inflammatory mechanism of depression, which journals they have published in, which institutions and individuals are playing a leading role in cooperation, and the dynamic changes of research direction. However, there are still several limitations. First, following several previous studies [30, 118], the literature search was limited to the WOSCC database so we may have missed some literature in other databases. Second, the literature we searched and included was mainly in English. As mentioned above, China is publishing a large number of research papers in this field with a burst trend, so we might have ignored literature in other languages (especially Chinese). Third, due to the expression of the abbreviations, the analysis results regarding the authors may not be solid enough. For example, the studies of Wang Y and Zhang Y may include many different authors.

## CONCLUSION

In conclusion, this study systematically evaluated the progress of research on the relationship between depression and inflammation by classifying and summarizing the literature from 1990 to 2023. It can be found that the inflammatory mechanism of depression has received extensive attention. The number of papers published in this field is increasing year by year, with significant contributions from the United States and mainland China. Regarding the cooperation between countries/regions, the United States also plays a leading role and has the highest centrality in the cooperation network. Among 91,100 authors, Maes M has the largest number of papers and co-citations at the same time. According to the keywords burst and co-cited reference analysis, the hotspots in the field in recent years are kynurenine, CRP, neuroinflammation, and gut microbiota. These directions may be the rising and promising research areas in the near future. Furthermore, there are also emerging clinical studies exploring the feasibility of using inflammation as a biomarker for the diagnosis, classification, targeted treatment, and prognosis prediction of depression. Finally, international academic exchanges and cooperation should be strengthened to promote breakthroughs in this field.

## Figures and Tables

**Fig. (1):**
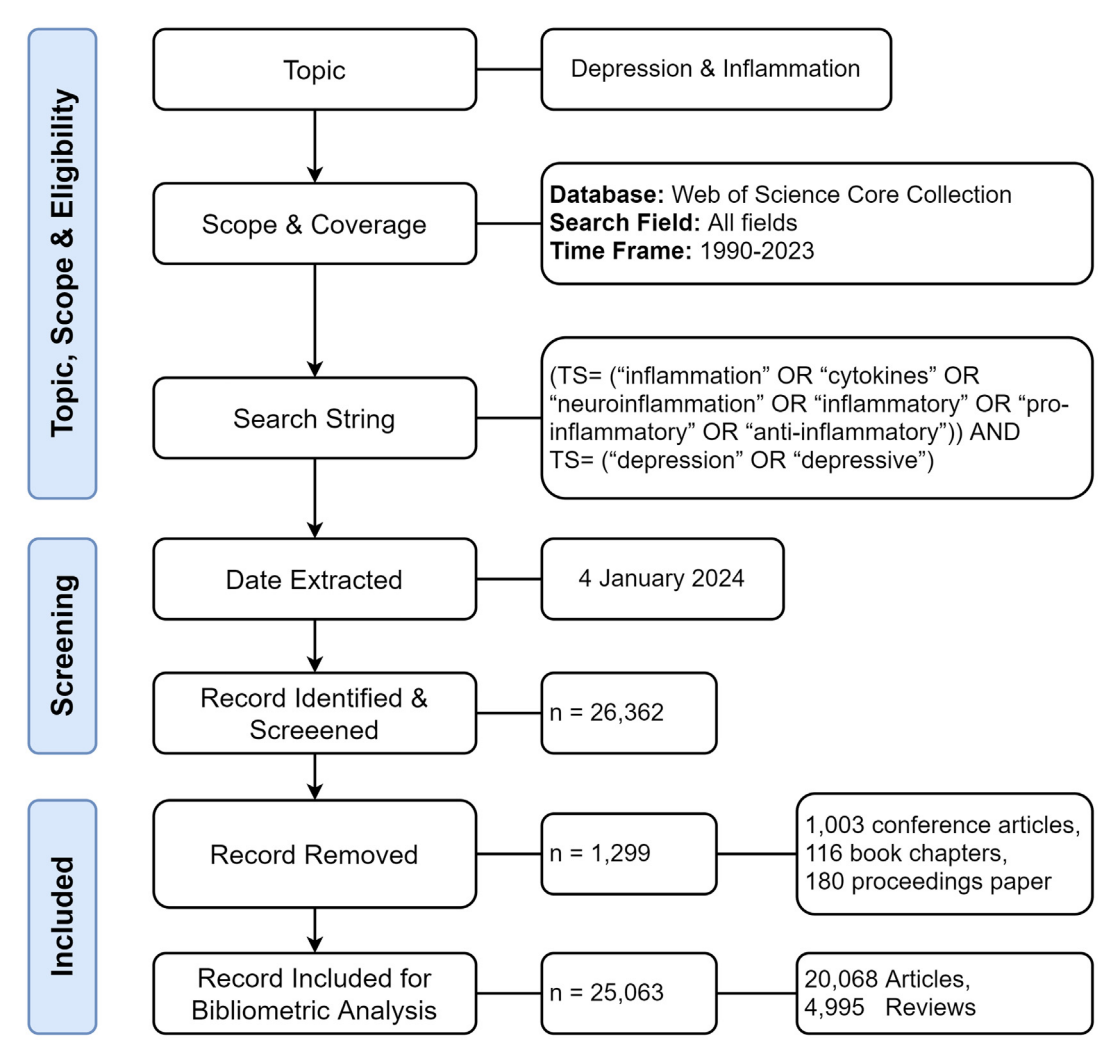
Flowchart for including and excluding literature studies. The flowchart shows the process of literature inclusion and exclusion.

**Fig. (2):**
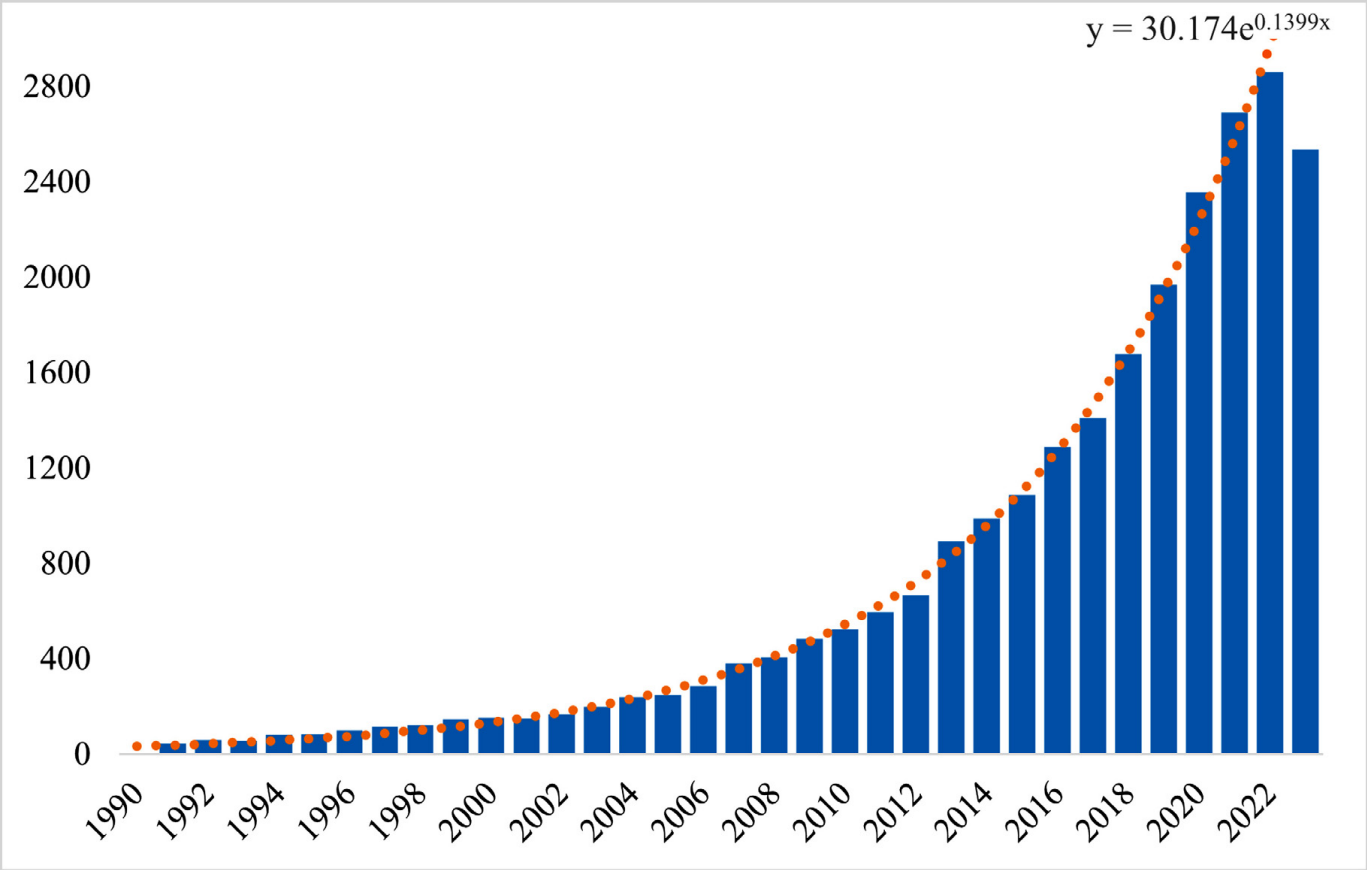
Annual publication on the topic of depression and inflammation over the past 30 years. The graph shows the number of publications in this field from 1990 to 2023. The bar represents the number of publications, and the dotted line indicates the change in the number of publications.

**Fig. (3):**
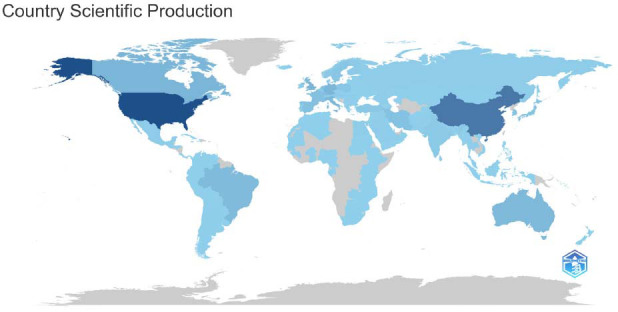
Geographical distribution of publications. The map shows the number of publications in the field by country from 1990 to 2023. Darker colors represent more publications, while lighter colors represent fewer publications.

**Fig. (4):**
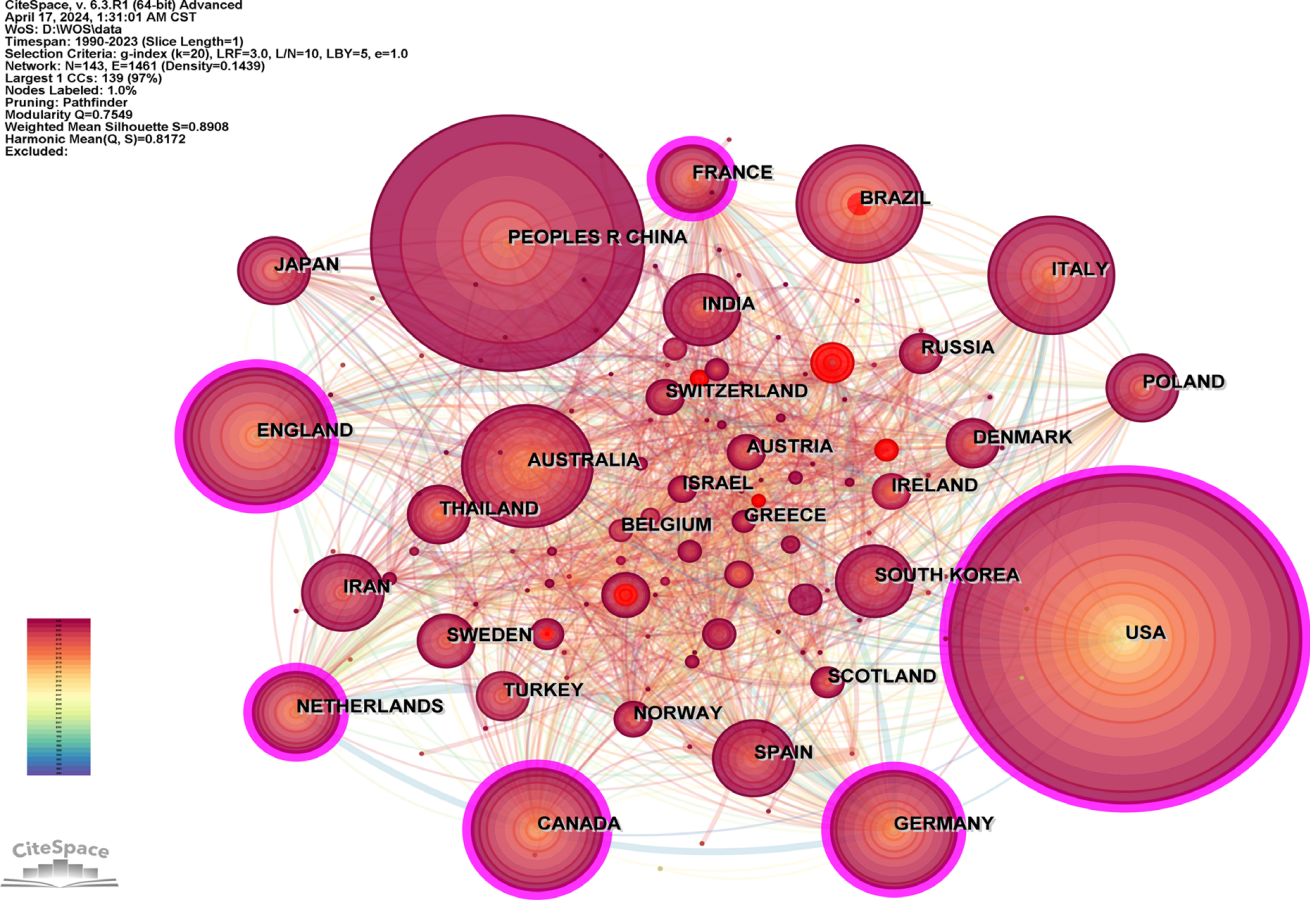
shows the cooperation between countries/regions, which has 143 nodes and 1461 connections with a network density of 0.1439. The larger the node, the more the papers were published. The change in node color indicates the time of publishing. The larger the centrality of a node is, it means that the node is in a leading position in the cooperative network. As shown in Fig. (**4**), the USA (publication: 7775, centrality: 0.14) has the highest publications and centrality, which means that the USA is the leading country in investigating the relationship between depression and inflammation. Specifically, since 1990, the USA has been involved in international efforts to study the inflammatory mechanism of depression. In 2023 alone, the USA contributed 543 collaborative papers. Subsequently, the People's R China (publication: 4252, centrality: 0.01) ranks second in publication, but the centrality is only 0.01. This demonstrates that the international cooperation of China in this field has not yet formed sufficient influence. The results also show that China mainland entered this field relatively late and did not publish its first international cooperation paper in this field until 2007. Nevertheless, China exhibits the highest burst rate, indicating that its influence in this field has gradually increased in recent years. The Figure shows the cooperation between countries. The size of the node reflects the number of publications in the country/region, and the color represents the time of publication. Specifically, red color indicates that the publishing time is closer to the present, while purple color indicates that the publishing time is earlier. A node with a purple circle indicates that the node has high centrality. These lines represent links between two different nodes.

**Fig. (5):**
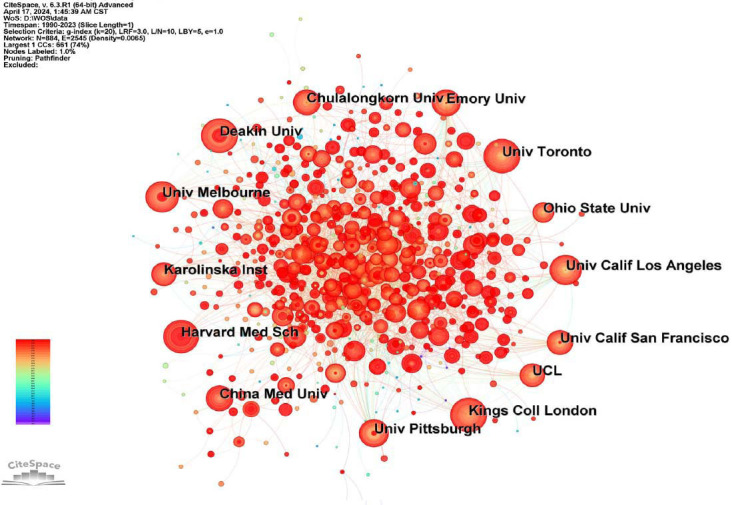
Collaborations among institutions. The Figure shows the cooperation between institutions, the size of nodes shows the number of publications in the institution, and the color represents the time of publication. Specifically, the red color means the time of publication is closer to the present, while the dark purple color means that the time of publication is earlier. These lines represent the link between two different nodes.

**Fig. (6):**
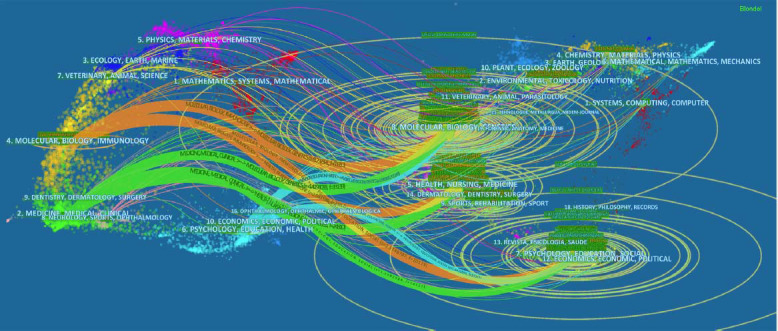
The dual-map overlay of journals on the “inflammatory mechanism of depression”. The Figure shows the relationship between citing and cited literature in journal classification. The citing journals are on the left, the cited journals are on the right, and the colored path represents the citation relationship.

**Fig. (7):**
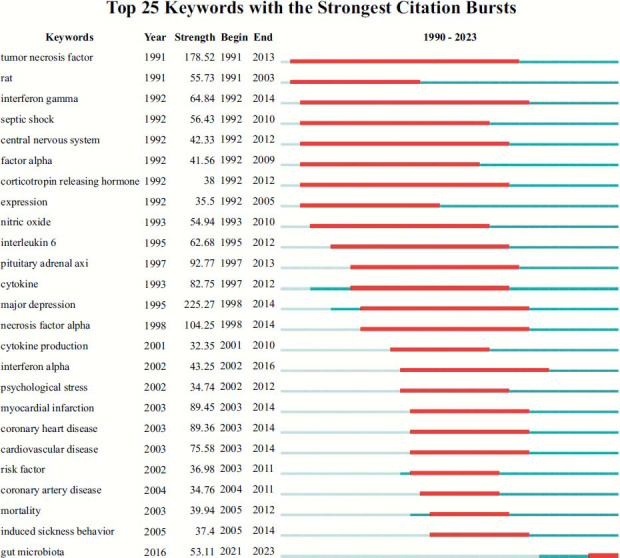
Top 25 keywords with strongest citation bursts. Bursts represent the strength and intensity of keyword bursts, which provides a useful means for tracking the development of keyword research. The blue thin line shows the entire period from 1990 to 2023. The position and length of the red line indicate the start and end time of the whole burst cycle and the duration of the burst.

**Fig. (8):**
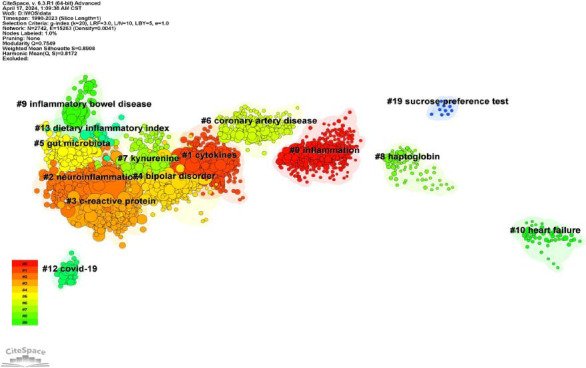
Reference co-citation clusters. The Figure shows the clustering of co-cited literature and the relationship between clusters. The name of each cluster is marked with a black label, representing the focus in this research field at that time.

**Fig. (9):**
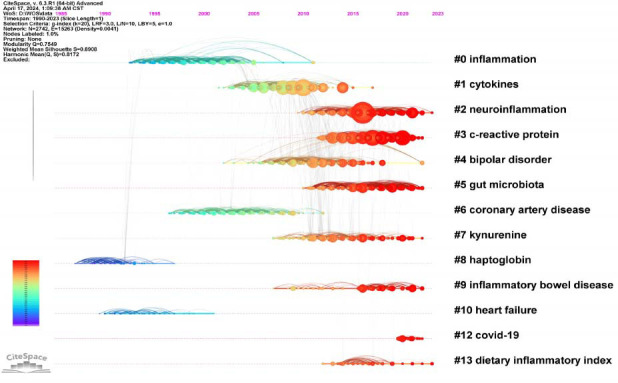
Timeline view of the temporal progression of activity in each cluster. The circle represents the co-cited literature. The time zone of the circle is the year when the literature was first published in this research cluster. The radius of the circle represents the frequency of occurrence, the color represents different publication times, and the line represents the connection between clusters. The time zone diagram shows the evolution of research hotspots in this field.

**Fig. (10):**
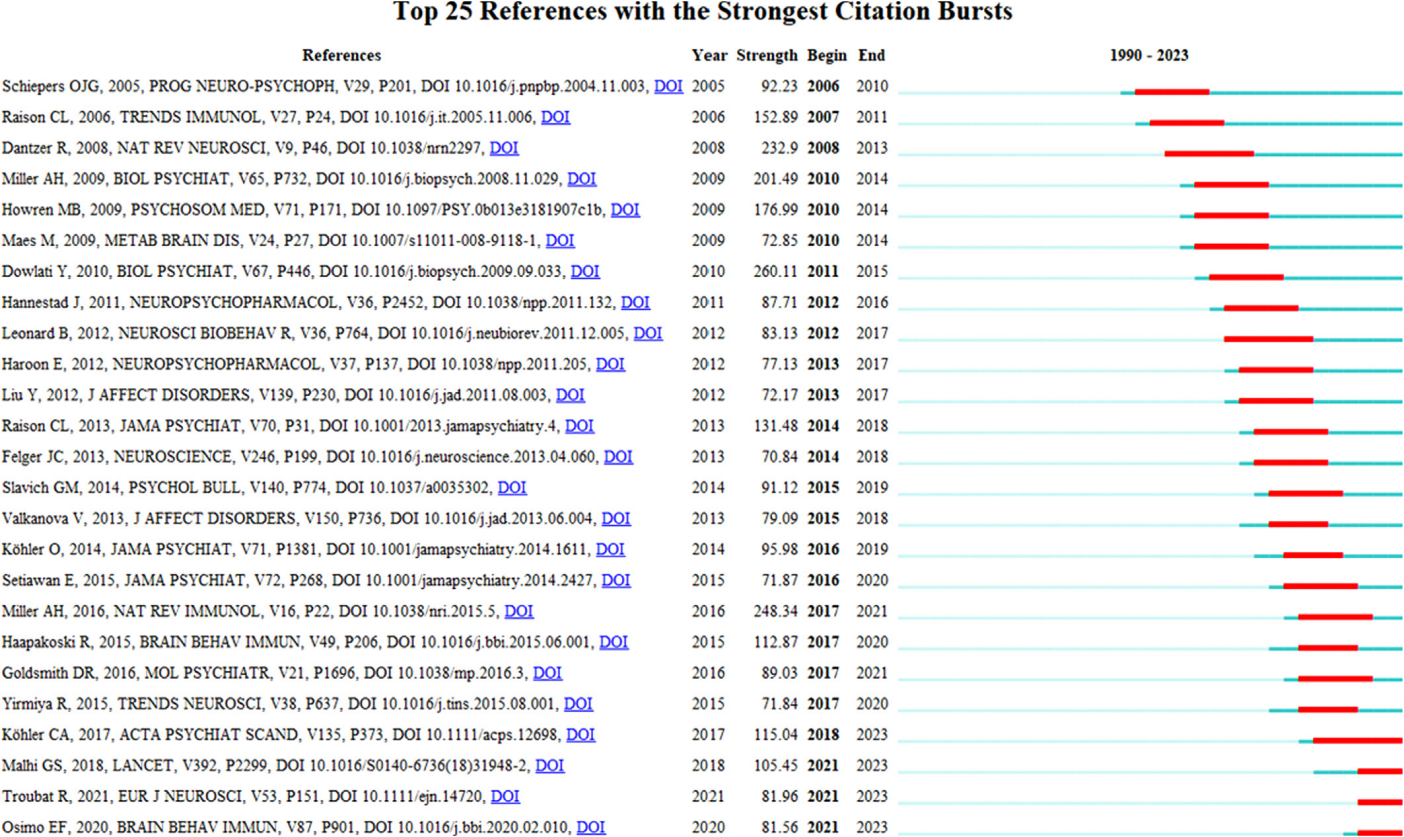
Top 25 references with the strongest citation bursts. Burst indicates citation burst strength. The blue bar shows the reference had been published, while the red bar represents citation burstness.

**Table 1:** Top 10 countries and institutions in terms of publications and centrality in collaboration network.

**No.**	**Country**	**Publication**	**Centrality**	**Burst**	**Institution**	**Publication**	**Centrality**	**Burst**
1	USA	7775	0.14	4.69	University of Toronto (Canada)	445	0.04	9.75
2	Peoples’ R China	4252	0.01	359.32	Kings College London (UK)	431	0.04	9.68
3	UK	1889	0.14	3.95	University of California, Los Angeles (USA)	383	0.02	27.34
4	Canada	1635	0.12	12.79	Deakin University (Australia)	354	0.01	11.9
5	Germany	1628	0.10	30.94	University of Melbourne (Australia)	320	0.02	9.89
6	Italy	1438	0.09	6.38	Emory University (USA)	279	0.04	26.17
7	Australia	1284	0.07	5.71	Harvard Medical School (USA)	278	0.00	17.91
8	Brazil	1169	0.04	8.03	University of Pittsburgh (USA)	251	0.02	20.21
9	Netherlands	1021	0.13	24.08	University College London (UK)	230	0.01	12.46
10	France	791	0.11	15.13	Ohio State University (USA)	241	0.01	21.32

**Table 2:** Top 10 authors and co-cited authors.

**No.**	**Author**	**Count (%)**	**Co-cited Author**	**Frequency**	**Centrality**
1	Maes M	608 (2.43%)	Maes M	4026	0.16
2	Berk M	350 (1.40%)	Dantzer R	3521	0.13
3	Zhang Y	307 (1.22%)	Miller AH	2989	0.02
4	Pariante CM	271 (1.08%)	Raison CL	2696	0.01
5	Miller AH	251 (1.00%)	Dowlati Y	2081	0.01
6	Mcintyre RS	214 (0.09%)	Capuron L	1558	0.01
7	Carvalho AF	192 (0.08%)	Howren MB	1249	0.00
8	Dantzer R	157 (0.06%)	Kessler RC	1216	0.00
9	Irwin MR	155 (0.06%)	Felger JC	981	0.00
10	Galecki P	149 (0.05%)	Beck AT	953	0.10

**Table 3:** Top 10 journals and co-cited journals.

**No.**	**Journal**	**Count (%)**	**IF (2023)**	**JCR**	**Co-cited Journal**	**Citation**	**IF (2023)**	**JCR**
1	Brain Behavior and Immunity	974 (3.43%)	15.1	Q1	Brain Behavior and Immunity	10757	15.1	Q1
2	Journal of Affective Disorders	500 (1.76%)	6.6	Q1	Biological Psychiatry	10334	10.6	Q1
3	Biological Psychiatry	498 (1.75%)	10.6	Q1	PLOS ONE	9615	3.1	Q2
4	Plos One	358 (1.26%)	3.1	Q2	Journal of Affective Disorders	8582	6.6	Q1
5	Psychoneuroendocrinology	352 (1.24%)	3.7	Q2	Proceedings of the National Academy of Sciences of the United States of America-Physical Sciences	8507	11.1	Q1
6	International Journal of Molecular Sciences	346 (1.22%)	5.6	Q1	Molecular Psychiatry	7833	11.0	Q1
7	Neuropsychopharmacology	345 (1.21%)	7.6	Q1	LANCET	7516	168.9	Q1
8	European Neuropsychopharmacology	313 (1.10%)	5.6	Q1	NATURE	6642	64.8	Q1
9	Frontiers in Psychiatry	312 (1.10%)	4.7	Q2	Neuropsychopharmacology	6628	7.6	Q1
10	Scientific Reports	244 (0.86%)	4.6	Q2	Progress In Neuro Psychopharmacology Biological Psychiatry	6487	5.6	Q1

**Table 4:** Top 6 references in structure variations analysis.

**Title**	**Publication Type**	**First Author**	**Publication Year**	**Journal**	**Modularity Change Rate**	**Citations**	**Cluster Linkage**	**Centrality Divergence**
Effects of pregnancy and delivery on the availability of plasma tryptophan to the brain: Relationships to delivery- induced immune activation and early post-partum anxiety and depression	Article	Michael Maes	2001	Psychological Medicine	98.43	57	46.39	0.21
The immunoregulatory effects of antidepressants	Review	Michael Maes	2001	Human Psychopharmacology	97.75	127	55.14	0.3
Paroxetine for the prevention of depression induced by high- dose interferon alfa	Clinical Trial	Dominique L. Musselman	2001	New England Journal of Medicine	96.92	744	-8.9	0.16
Plasma inflammatory cytokine response to surgical trauma in chronic depressed patients	Article	Akira Kudoh	2001	Cytokine	96.92	23	-10.09	0.15
Aging exacerbates depressivelike behavior in mice in response to activation of the peripheral innate immune system	Article	Jonathan P Godbout	2008	Neuropsychopharmacology	96.2	200	-5.11	0.02
Inflammation, glutamate, and glia in depression: A literature review	Review	Leah McNally	2008	CNS Spectrum	96.2	218	-3.48	0.01

**Table 5:** Characteristics of key studies on Inflammatory mechanisms of depression.

Study Type	Theory/Therapy	Main Findings	References
Basic study	BBB opening	Disrupted BBB integrity mediated in part by TNF-a contributes to blocking recovery from prolonged learned helplessness depression-like behavior	Chen *et al.,* (2018) [71]
Basic study	Gut-brain axis	Fecal microbiota transplantation of GF mice with 'depression microbiota' derived from MDD patients resulted in depression-like behaviors compared with colonization with 'healthy microbiota' derived from healthy control individuals.	Zheng *et al.,* (2016) [72]
Basic study	IDO/Kynurenine Pathway	Administration of L-kynurenine elicited depressive-like behaviors, whereas the depressive-like behaviors induced by LPS were attenuated by an IDO competitive inhibitor	O'Connor *et al.,* (2009) [73, 74]
Basic study	Inflammasome	The acute restraint stress rapidly induced an increase in extracellular ATP, which acts as an endogenous agonist of P2X7R. Additionally, it led to elevated levels of the inflammatory cytokine IL-1β and the active form of the NLRP3 inflammasome within the hippocampus.	Iwata *et al.,* (2016) [75]
Basic study	Sympathetic nervous system	Sickness behavior, a physiological and behavioral response associated with heightened immune activity, is partially modulated by the vague nerve through interactions with immune cells.	Dantzer *et al.,* (2008) [43]
Clinical evidence	BBB opening	BBB permeability is negatively correlated with the severity of depressive symptoms	Niklasson *et al.,* (1984) [76]
Clinical evidence	Gut-brain axis	Gut microbiota potentially plays a pivotal role in the pathogenesis of depression, with dysbiosis of microbial communities being implicated in the manifestation of depression-like behaviors.	Rogers *et al.,* (2016) [77]
Clinical evidence	IDO/Kynurenine Pathway	The Kynurenine (Kyn) Pathway exhibits activation in individuals diagnosed with MDD.	Lin *et al.,* 2023 [78]
Clinical evidence	Inflammasome	The expression of NLRP3 and caspase-1 in circulating immune cells is upregulated in MDD patients, indicating an activated NLRP3 inflammasome, which correlates with elevated levels of IL-1β and IL-18 in the blood.	Alcocer-Gomez *et al.,* (2014) [79]
Clinical evidence	Sympathetic nervous system	The autonomic nervous system is altered in depression, with increased sympathetic activity and lower parasympathetic tone.	Murphy, (1991) [80]
Clinical practice	Mindfulness meditation	The implementation of a community-accessible mindful awareness practices intervention led to enhancements in sleep quality and reductions in TNF-α levels.	Black *et al.,* (2015) [81]
Clinical practice	Exercise intervention	High TNF-a may differentially predict better outcomes with exercise treatment as opposed to antidepressant medications for which high TNF-a is linked to poor response.	Rethorst *et al.,* (2013) [82]
Clinical practice	Drug intervention	Vortioxetine with celecoxib may potentiate the antidepressant effects while concurrently reducing levels of inflammatory cytokines	Sampson *et al.,* (2024) [83]

## Data Availability

The original contributions presented in the study are included in the article. Further inquiries can be directed to the corresponding authors.

## LIST OF ABBREVIATIONS

MDD: Major Depressive Disorder
SCI-EXPANDED: Science Citation Index Expanded
SSCI: Social Sciences Citation Index
CPCI-SSH: Conference Proceedings Citation Index- Social Science & Humanities
BKCI-S: Book Citation Index– Science
BKCI-SSH: Book Citation Index– Social Sciences & Humanities
WOSCC: Web of Science Core Collection
CCR-EXPANDED: Emerging Sources Citation Index (ESCI); Current Chemical Reactions
IC: Index Chemicus
PNIRS: Psychoneuroimmunology Research Society
HPA: Hypothalamic-Pituitary-Adrenal
